# Multilayer network analysis of mental health symptoms in UK University students: association patterns of depression, loneliness, and suicidal ideation

**DOI:** 10.3389/fpsyt.2026.1682965

**Published:** 2026-02-27

**Authors:** Xiao-Han Zhang, Heng Miao, Wen-Jing Yan, Tian-Tian Zheng, Hui-Zhen Lyu

**Affiliations:** 1 Renji College, Wenzhou Medical University, Wenzhou, China; 2 School of Mental Health, Wenzhou Medical University, Wenzhou, China; 3 Xi’an No. 3 Hospital, Xi’an, China

**Keywords:** depression, loneliness, network analysis, suicidal ideation, university students

## Abstract

Mental health problems among university students are increasingly severe, with symptoms such as depression, anxiety, loneliness, and suicidal ideation frequently co-occurring to form complex symptom networks. This study systematically analyzed association patterns of mental health symptoms among UK university students through multilayer network analysis. A cross-sectional survey was conducted with 1,285 students from five UK universities, who are assessed using eight validated psychometric instruments evaluating depression, anxiety, mania, sleep quality, stress, suicidal ideation, psychotic experiences, and loneliness. A dual-level network analysis approach was employed, constructing both a scale-level network with 8 nodes to identify macro-association patterns and an item-level network with 33 nodes for in-depth analysis of depression, loneliness, and suicidal ideation connections. The EBICglasso algorithm estimated network structure, and key symptoms were identified through centrality indices. The scale-level network revealed depressive symptoms as most prominent across all centrality indices, establishing their core position. The strongest connections existed between anxiety-depression (edge weight = 0.37) and anxiety-stress (edge weight = 0.35), while loneliness connected with psychotic experiences (edge weight = 0.23) and suicidal ideation (edge weight = 0.144). In item-level analysis, thoughts of death (PHQ_9), lack of companionship (UCLA3_4), and frequency of suicidal thoughts (SBQ2) demonstrated strongest bridge centrality. Network stability analysis showed CS coefficients reached the good standard of 0.5. These findings demonstrate that depressive symptoms occupy a core network position, loneliness plays a unique bridging role, and suicidal ideation closely associates with depression and loneliness, providing evidence for network-based precision intervention strategies.

## 1 Introduction

University students are facing unprecedented mental health challenges. According to the WHO World Mental Health International College Student Survey in 2018, approximately 35% of first-year university students across 19 countries had a lifetime history of one or more DSM-IV mental disorders, with a 12-month prevalence rate of 31% (1). These issues affect individual academic performance and quality of life while posing significant challenges to higher education systems. The complexity of university students’ mental health problems lies in the interaction of multiple symptom dimensions. Depression, anxiety, loneliness, and suicidal ideation frequently co-occur, forming complex symptom networks (2). Network analysis approaches treat psychological symptoms as interconnected network nodes, identifying direct associations and key symptom nodes through edge weights and network topology analysis, providing scientific evidence for precision intervention (3).

### 1.1 Theoretical foundations of mental health symptom network analysis

Network theory in psychopathology assumes mental disorders are maintained through direct causal interactions between symptoms rather than underlying common factors (4). Mutual activation between symptoms constitutes mental disorders’ essence, rather than symptoms being external disease manifestations. This framework provides new explanatory mechanisms for complex psychological phenomena, particularly in explaining symptom comorbidity patterns and treatment mechanisms. Within network analysis, centrality indices identify key symptoms. Strength centrality reflects connection strength between nodes, expected influence considers negative correlations’ impact, betweenness centrality measures nodes’ importance in network information transmission, and closeness centrality reflects a node’s ability to influence the entire network. These indices help identify influential symptoms and provide quantitative tools for understanding symptom transmission mechanisms.

### 1.2 Current state of network research on university students’ mental health symptoms

Existing network research primarily focuses on depression and anxiety symptom analysis. Research on Dutch university students found depression and anxiety symptoms showed high interconnectedness, with fatigue, worry, and loss of interest serving as key bridge symptoms (5). Studies among Belgian university students found “feeling sad” and “feeling nervous” had the highest centrality indices (6). However, current research is limited in symptom scope, with most studies focusing on only 2–3 symptom dimensions, lacking comprehensive analysis of the broader mental health spectrum. Additionally, existing research mainly employs scale-level analysis, lacking item-level fine-grained analysis to reveal internal symptom structures and cross-symptom micro-connection mechanisms.

### 1.3 Theoretical associations of key symptom dimensions

Depression symptoms typically occupy central positions in mental health networks. Cognitive triad theory emphasizes negative cognition’s role in depression development, with network analysis providing empirical support (7). Cognitive-related depression symptoms (worthlessness, hopelessness) often have high centrality (8), while somatic symptoms (fatigue, sleep problems) show important network positions (9). Loneliness may play a unique bridge role in mental health networks. Social pain theory indicates loneliness triggers and maintains various mental health problems (10). Theoretically, the association between loneliness and depression can be understood through several mechanisms. The Social Support Buffering Model suggests that loneliness represents a perceived lack of resources to cope with stressors, which facilitates the transition from acute stress to clinical depression (11, 12). Furthermore, from a neurobiological perspective, social pain theory posits that the distress of loneliness activates similar neural pathways to physical pain, creating a state of hyper-vigilance to social threats that fuels depressive cognitions (13). This constant state of ‘social threat’ may serve as the engine that activates other symptoms within the mental health network. A growing body of empirical evidence supports a bidirectional relationship between loneliness and depression. Existing research has confirmed that, on one hand, loneliness not only significantly increases the risk of depression but also further exacerbates depressive symptoms (14); on the other hand, this association exhibits robust bidirectional characteristics, whereby depression reciprocally intensifies an individual’s sense of loneliness, forming a vicious cycle (15). Within this bidirectional relationship, the pathway from loneliness to depression has received more extensive empirical support. The impact of loneliness on depression demonstrates consistency across different cultural contexts. Riboldi et al.’s (16) cross-cultural study identified significant correlations between loneliness and depressive symptoms in both Italian and British university student samples, establishing loneliness as a robust core predictor of depression among college students. More importantly, loneliness functions not only as an independent risk factor for depression but may also serve as a mediator in the relationship between stress and depression. Through mediation analysis, Riboldi et al. (17) revealed that among students experiencing traumatic or high-stress life events (such as university entrance), loneliness acted as a mediating variable in the transformation of traumatic stress responses into depressive symptoms, accounting for approximately one-third of the total effect. These findings further underscore the importance of addressing social connection in mental health interventions for college students and suggest that interventions targeting loneliness may represent an effective approach to preventing and alleviating depressive symptoms.

Network analysis shows loneliness has direct associations with multiple symptom dimensions including depression, anxiety, and suicidal ideation, potentially becoming an important transmission pathway (18). Suicidal ideation positioning in networks has significant clinical implications. Joiner’s interpersonal-psychological theory emphasizes thwarted belongingness, perceived burdensomeness, and acquired capability roles in suicide risk (19). Research indicates hopelessness and worthlessness show strong network connections with suicidal ideation (20), while social isolation and loneliness may bridge other symptoms with suicidal ideation (21). The focus on depression, loneliness, and suicidal ideation is grounded in Joiner’s Interpersonal-Psychological Theory of Suicide (IPTS) (19). According to IPTS, suicide risk emerges from thwarted belongingness, perceived burdensomeness, and acquired capability. Loneliness directly mirrors thwarted belongingness, while depressive symptoms align with perceived burdensomeness.

### 1.4 The present study

Although mental health symptom network analysis has become important in psychopathology research, existing studies have significant limitations. Most studies focus on limited symptom dimensions, lacking comprehensive analysis of university students’ mental health problems. Research often concentrates on depression-anxiety binary models (22, 23), ignoring other important dimensions. Beyond the limited coverage of symptom dimensions, the absence of analytical granularity constitutes another critical gap: existing research predominantly employs scale-level analyses, treating complex constructs such as loneliness and depression as unitary entities. This approach fails to reveal fine-grained ‘micro-connections’ between specific symptoms—for instance, how particular facets of loneliness (e.g., lack of companionship) may disproportionately drive thoughts of self-harm. By adopting a multilayer network approach that bridges macro-level associative patterns with item-level granularity, This study aims to construct a comprehensive symptom network containing eight dimensions--depression, anxiety, mania, sleep quality, stress, suicidal ideation, psychotic experiences, and loneliness--to systematically analyze association patterns among UK university students through multilayer network analysis. The present study aims to identify specific ‘bridge symptoms’ that serve as primary conduits for comorbidity and suicidal risk in the UK university population. This study employs a multilayer network approach, ‘multilayer’ refers to analyses at two granularities (scale and item) rather than a multiplex model with explicit inter-layer edges. This approach offers unique insights by pinpointing not only which disorders are central to university students’ distress but also the specific ‘bridge symptoms’—such as death-related cognitions—that drive the transition between general depression and active suicidal ideation. The selection of eight symptom dimensions aims to overcome the limitations of narrow binary models and provide a comprehensive map of student mental health. Within this broad network, we specifically prioritized depression, loneliness, and suicidal ideation for item-level analysis. This focus is theoretically grounded in the Interpersonal-Psychological Theory of Suicide (IPTS), which posits that the interaction between interpersonal deficits (loneliness) and depressive cognitions (burdensomeness) is critical for understanding suicide risk. Specific objectives include: (1) constructing a scale-level multilayer network model to identify key symptom dimensions; (2) conducting item-level network analysis of depression, loneliness, and suicidal ideation; (3) providing scientific evidence for precise identification and effective intervention.

## 2 Method

### 2.1 Participants and procedures

This study constituted a secondary analysis of a publicly available dataset. The original data collection was conducted as a cross-sectional online survey of UK university students from September 17, 2018, to July 30, 2019.The study adhered to British Psychological Society ethical standards and was approved by Sheffield Hallam University’s Research Ethics Committee (Protocol Number: ER7368595). Participants were recruited from five UK universities (Durham University, University of Glasgow, Northumbria University, Sheffield Hallam University, and University of Sheffield) through institutional course research credit schemes, faculty social media pages, and faculty emails. This dataset was selected due to its comprehensive inclusion of eight validated psychometric scales, which provides a unique opportunity to explore macro- and micro-level symptom interrelationships across a broad spectrum of mental health challenges.

The final sample included 1,408 participants aged 18–56 years (M = 20.94, SD = 4.42, completion rate 76.5%). The sample comprised 83% female (n = 1,169) and 16.8% male (n = 238). Most participants were undergraduate students (86.2%, n = 1,213), followed by taught postgraduate students (7.4%, n = 104). Regarding ethnicity, 79.2% were White British (n = 1,115). All participants provided informed consent.

### 2.2 Measures

This study employed eight validated psychometric instruments to assess mental health symptoms. The detailed psychometric properties, scoring criteria, and cut-off values for all scales have been comprehensively described and validated in the study by Akram et al. (24). The following provides a brief overview of the main characteristics of each scale:

Anxiety was measured using the 7-item Generalized Anxiety Disorder scale (GAD-7), with total scores ranging from 0-21, where higher scores indicate greater anxiety severity. The internal consistency reliability in this study was α = 0.923.

Depression was assessed using the 9-item Patient Health Questionnaire (PHQ-9), with scores ranging from 0-27, where higher scores reflect more severe depressive symptoms. The internal consistency reliability was α = 0.903.

Manic episodes were evaluated using the Mood Disorder Questionnaire (MDQ), with scores ranging from 0-13, where higher scores suggest more pronounced manic symptoms. The internal consistency reliability in this study was α = 0.821.

Sleep quality was measured using the 8-item Sleep Condition Indicator (SCI), with scores ranging from 0-32, where lower scores indicate more severe insomnia symptoms. The internal consistency reliability in this study was α = 0.861.

Stress was assessed using the 10-item Perceived Stress Scale (PSS), with scores ranging from 0-40, where higher scores reflect greater perceived stress. The internal consistency reliability in this study was α = 0.865.

Suicidal ideation was evaluated using the 4-item Suicidal Behaviors Questionnaire-Revised (SBQ-R), with scores ranging from 3-18, where higher scores indicate higher suicide risk. The internal consistency reliability in this study was α = 0.840.

Psychotic experiences were assessed using the 16-item Prodromal Questionnaire (PQ-16), with scores ranging from 0-16, where higher scores indicate more frequent psychotic-like experiences. The internal consistency reliability in this study was α = 0.816.

Loneliness was measured using the 20-item UCLA Loneliness Scale Version 3 (UCLA-3), with scores ranging from 20-80, where higher scores reflect stronger feelings of loneliness. The internal consistency reliability in this study was α = 0.935.

### 2.3 Data analysis

This study employed dual-level network analysis to understand mental health symptom interrelationships at different granularity levels. First, a scale-level network (8 nodes) explored macro-association patterns among mental health dimensions. Second, an item-level network focused on symptom items from depression, loneliness, and suicidal ideation (33 nodes) to understand micro-connection mechanisms within and between these key symptom clusters. Listwise deletion handled missing data to ensure complete case analysis. Descriptive statistics were calculated for all mental health indicators. Mental health symptom networks were constructed using qgraph and bootnet packages in R software (version 4.3.0). Networks were estimated using the Extended Bayesian Information Criterion graphical least absolute shrinkage and selection operator (EBICglasso) algorithm, which employs regularization methods to identify meaningful connections while removing spurious edges. In scale-level analysis, each mental health dimension was represented as a network node. In item-level analysis, each item from depression, loneliness, and suicidal ideation scales was represented as a node. Edges represent partial correlations between symptoms after controlling for all other network variables. For scale-level networks, four centrality indices were calculated: strength centrality (sum of absolute edge weights), expected influence (sum of all edge weights including negative weights), betweenness centrality (number of shortest paths between nodes passing through a given node), and closeness centrality (reciprocal of sum of shortest paths from a node to all others). For item-level networks, bridge centrality analysis identified symptoms playing important connecting roles between symptom domains. Network stability was assessed through bootstrap procedures. Case-dropping bootstrap with 1,000 samples evaluated network stability, calculating correlation stability coefficients (CS-coefficient) for centrality indices. Values ≥0.25 were acceptable, ≥0.5 were good. All analyses used bootnet, qgraph, networktools, psych, and igraph packages in R (version 4.3.0). Statistical significance was set at α = 0.05. In addition to network estimation, Multidimensional Scaling (MDS) was used to visualize the proximities between variables. While the EBICglasso identifies direct edges between symptoms after controlling for other variables, MDS provides a complementary perspective by mapping nodes into a low-dimensional space where distance reflects the strength of their zero-order associations, helping to identify latent symptom clusters.

## 3 Results

### 3.1 Sample description

The final sample consisted of 1,408 participants, with 123 participants excluded due to missing data handling, primarily due to incomplete questionnaires or missing key variables. The basic characteristics (such as gender and age distribution) of the excluded sample showed no significant differences from the final sample. A total of 1,285 participants were included in the analysis, with a mean age of 20.81 years (SD = 4.22), comprising 1,058 females (82.3%) and 227 males (17.7%). By course type distribution, undergraduate students comprised the largest proportion (1,118 participants, 87.0%), followed by taught postgraduate students (93 participants, 7.2%), doctoral students (32 participants, 2.5%), research postgraduate students (19 participants, 1.5%), and other types of students (23 participants, 1.8%).

### 3.2 Scale-level network analysis

**Figure 1:**
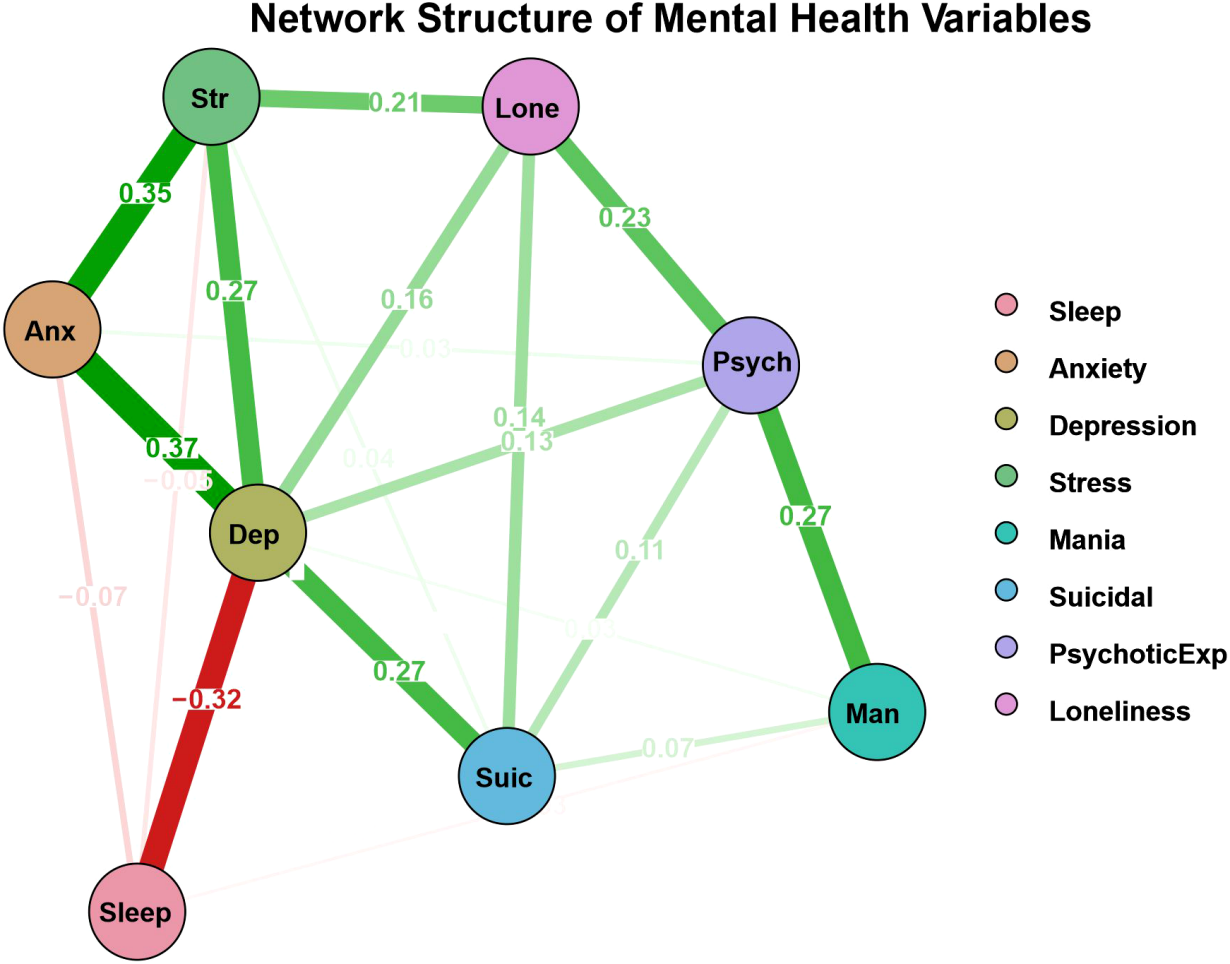
Scale-level mental health symptom network analysis results. The network structure is an EBICglasso network based on GGM. Green edges represent positive correlations, and red edges indicate negative correlations. The thickness of the edges reflects the magnitude of the correlations. It is important to note that the node positions do not indicate Euclidean distances. The values (weights) on the edges represent regularized regression coefficients.

The scale-level network analysis constructed a mental health symptom network comprising 8 nodes with 23 non-zero edge connections (**Figure 1**). The network exhibited a moderately sparse structure (82.1% connection density), with edge weights ranging from -0.321 to 0.372 and an average absolute edge weight of 0.138. Among symptom connections, the strongest positive connections appeared between anxiety and depression (edge weight = 0.37), and between anxiety and stress (edge weight = 0.35), reflecting the high comorbidity of these internalizing disorder symptoms. Sleep quality showed a significant negative association with depressive symptoms (edge weight = -0.32), indicating that sleep quality is closely related to the severity of depressive symptoms. Loneliness demonstrated important connection patterns in the network, establishing strong associations with psychotic experiences (edge weight = 0.23) and stress levels (edge weight = 0.211). Notably, the direct edge between loneliness and suicidal ideation remains statistically significant (edge weight = 0.144) after controlling for all other symptoms, including depression, suggesting that loneliness may function as an auxiliary or complementary contributor to suicide risk. Suicidal ideation showed a strong association with depressive symptoms (edge weight = 0.267), consistent with the central role of depressive symptoms in suicide risk assessment. Node connectivity analysis revealed that depression and suicidal ideation nodes had the highest connectivity (7 edges each), indicating that they occupy central positions in this mental health network.

**Figure 2:**
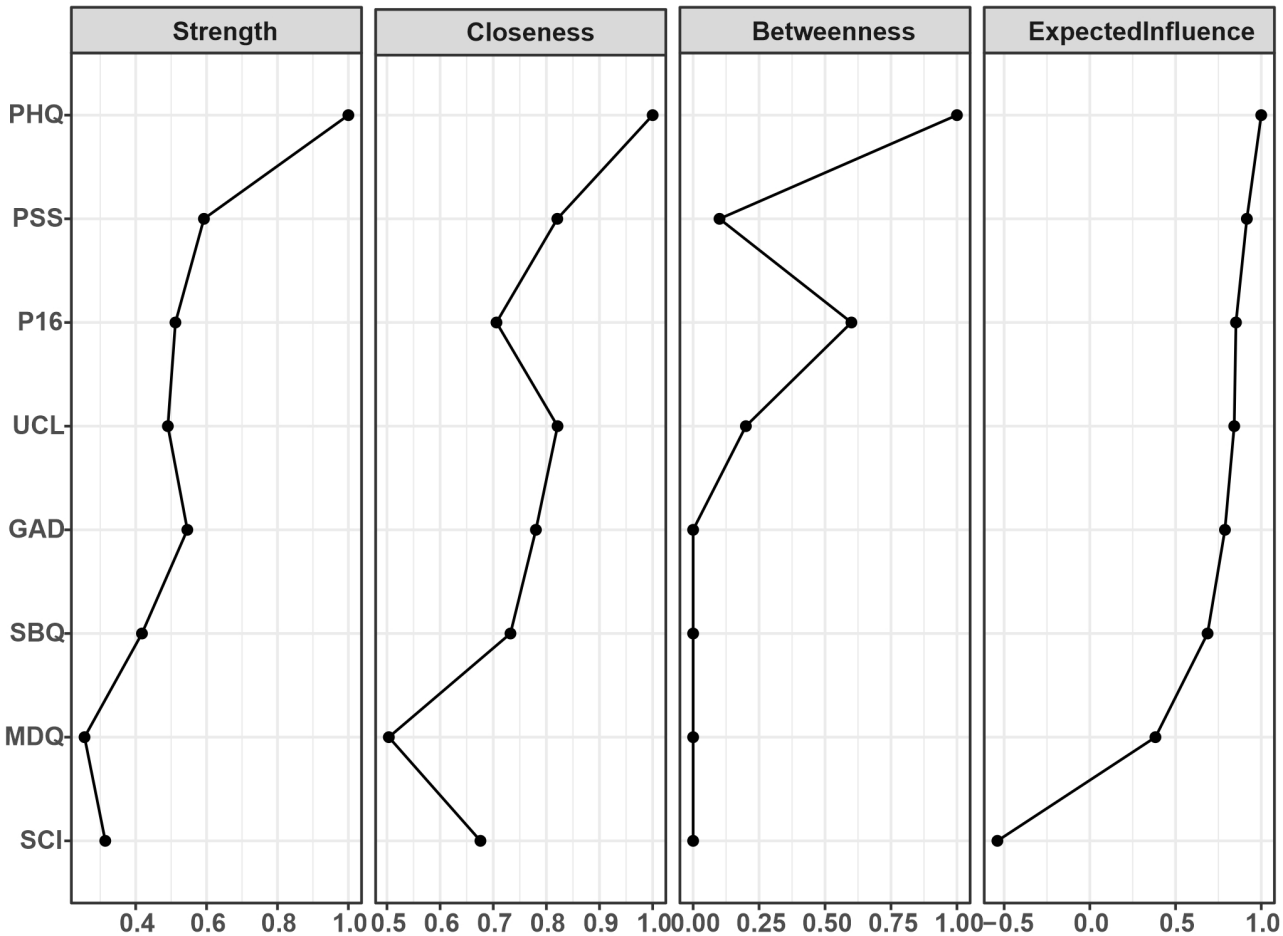
Centrality indices for the nodes of the present network, including those for strength, betweenness, closeness, and expected influence.

The centrality indices were all normalized (i.e., normalized relative to the highest value within each measure). The table presents centrality index results ranked by “Expected Influence” (**Figure 2**). Among all symptoms, depressive symptoms showed the most prominence across all four indices, demonstrating its central position in the entire network. Stress symptoms ranked second, indicating their important role in network connectivity. In terms of “closeness centrality” and “betweenness centrality,” psychotic experiences scored highly on betweenness centrality, playing an important bridging role and frequently positioned on critical pathways between other nodes. Loneliness symptoms and stress symptoms ranked highly in closeness centrality, indicating that they are relatively close to other nodes in the network and can influence multiple mental health symptoms through shorter pathways.

### 3.3 Item-level network analysis: depression-loneliness-suicidal ideation

**Figure 3:**
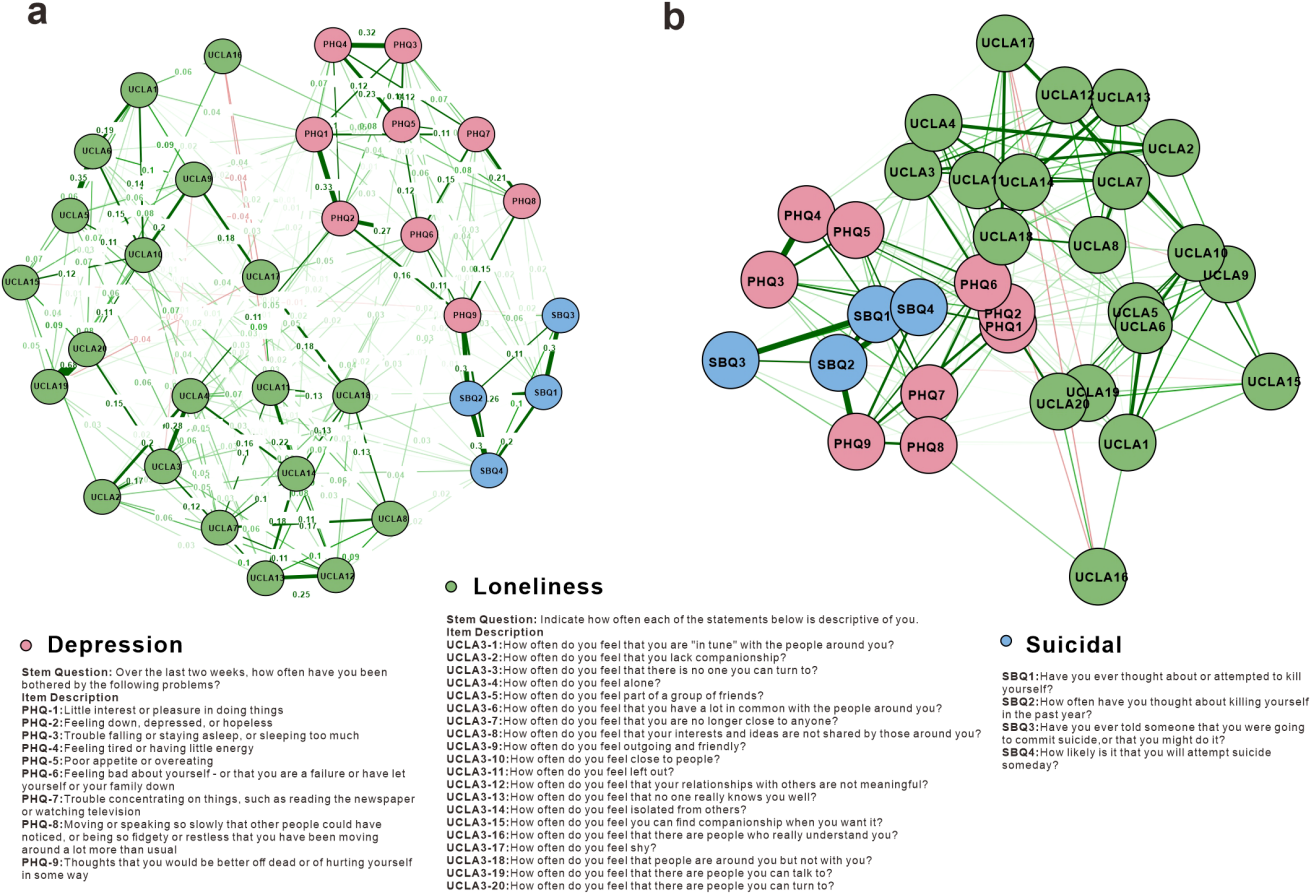
Item-level mental health symptom network analysis results. The network structure is an EBICglasso network based on GGM. Green edges represent positive correlations, and red edges indicate negative correlations. The thickness of the edges reflects the magnitude of the correlations. **(a)** The network is with “circle” layout for easy viewing. It is important to note that the node positions do not indicate Euclidean distances. The values (weights) on the edges represent regularized regression coefficients. **(b)** The network is with MDS, showing proximities among variables as distances between points in a low-dimensional space.

To gain in-depth understanding of the intrinsic mechanisms of three core symptom dimensions—depression, loneliness, and suicidal ideation—we constructed a detailed network comprising 33 symptom items. This network contained 211 non-zero edge connections (**Figure 3a**), exhibiting a moderately sparse structure with edge weights ranging from -0.041 to 0.681 and an average absolute edge weight of 0.072.

Within depression symptom internal connections, the strongest edge connections emerged between PHQ_1 (lack of interest or pleasure) and PHQ_2 (feeling down, depressed, or hopeless) (edge weight = 0.327), as well as between PHQ_3 (trouble falling asleep, staying asleep, or sleeping too much) and PHQ_4 (feeling tired or having little energy) (edge weight = 0.320). The UCLA loneliness scale demonstrated robust internal connectivity, with the most prominent being the strong association between UCLA3_19 (feel there are people I can talk to) and UCLA3_20 (feel there are people I can turn to) (edge weight = 0.681), both being reverse-coded items assessing social connection. Suicide-related items showed expected strong inter-correlations, with the most significant connection between SBQ1 (ever thought about or attempted suicide) and PHQ_9 (thoughts that you would be better off dead or hurting yourself) (edge weight = 0.304), reflecting the conceptual overlap between suicidal ideation and death-related cognitions in depression.

Multidimensional scaling analysis revealed (**Figure 3b**) that nodes within the three major symptom groups of depression, loneliness, and suicidal ideation were more tightly connected internally, displaying relatively independent clustering structures among the three domains. Within the depression symptom group, PHQ1-PHQ9 nodes formed tight clusters located in the left region of the figure. The loneliness symptom group (UCLA1-UCLA20) was primarily distributed in the central and upper-right regions of the figure, with UCLA19 and UCLA20 as reverse-coded items positioned close together, while UCLA15 and UCLA16 were positioned farther from other loneliness nodes. The suicidal ideation group (SBQ1-SBQ4) formed a relatively independent small cluster located in the lower-right region of the figure. Throughout the entire network, PHQ9 (thoughts of death) was positioned close to SBQ-related nodes, consistent with the conceptual overlap between death-related cognitions in depression and suicidal ideation.

**Figure 4:**
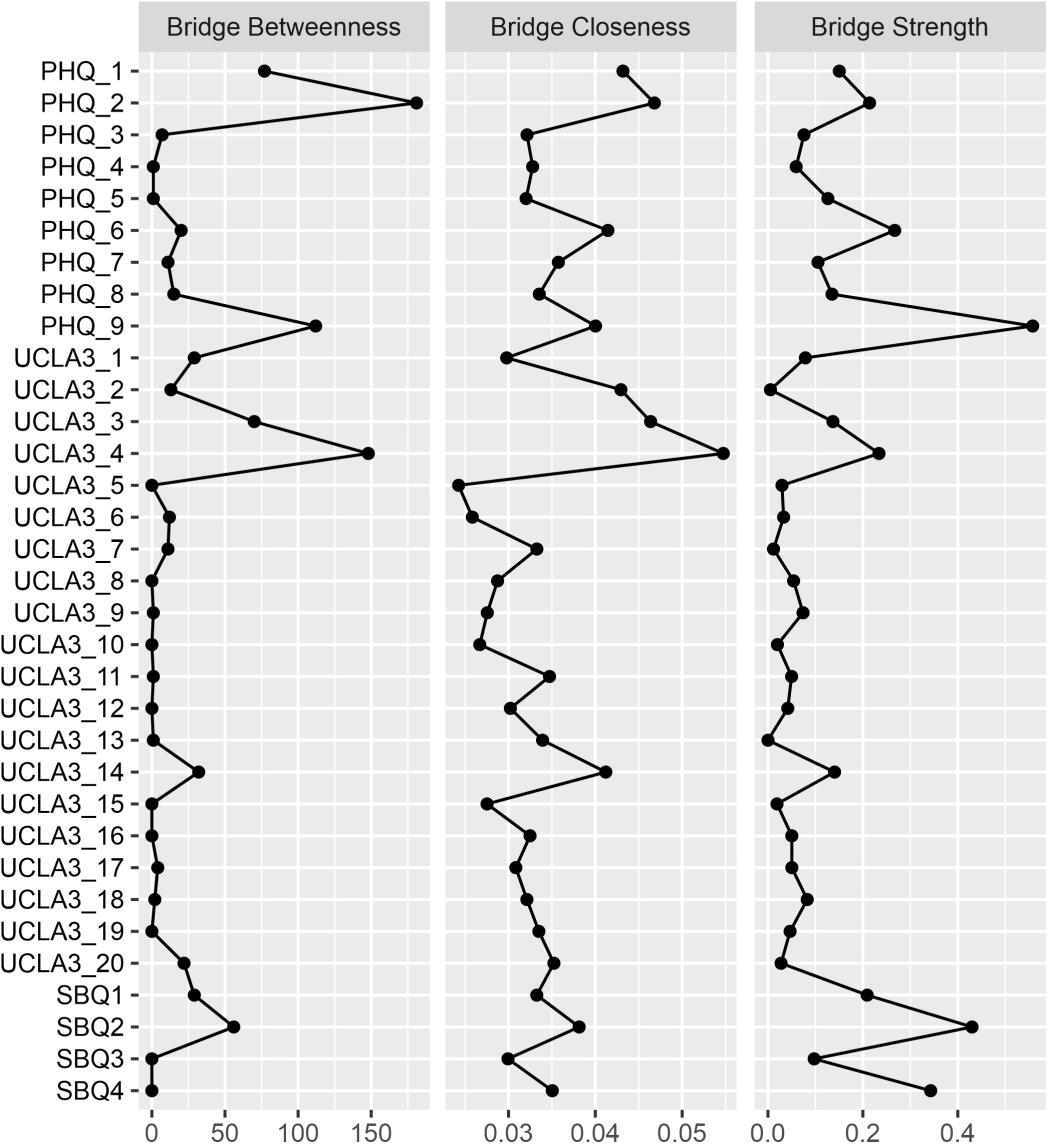
Estimated bridge centrality indices for the present network, including bridge strength, betweenness and closeness.

Bridge centrality analysis identified symptoms that play important connecting roles between different symptom domains(**Figure 4**). Among depression symptoms, PHQ_9 (thoughts of death or self-harm) ranked highest across all bridge centrality indices, indicating that this node has strong connections with both loneliness and suicidal ideation symptoms. PHQ_6 (feeling like a failure or letting yourself or family down) and PHQ_2 (feeling down, depressed, or hopeless) also demonstrated strong bridging relationships with these two symptom categories. Among loneliness symptoms, UCLA3_4 (lack companionship) performed prominently across multiple bridge centrality indices, being one of the loneliness items with the strongest connections to other categories. UCLA3_14 (feel isolated from others) and UCLA3_3 (feel left out) also showed prominence across multiple bridge centrality indices. Among suicidal ideation symptoms, SBQ2 (frequency of suicidal thoughts in the past year) and SBQ4 (likelihood of future suicide) were the suicidal ideation items with the strongest connections to other categories, particularly showing significance in bridge strength and bridge expected influence indices, demonstrating their closest bridging relationships with depression and loneliness symptoms.

Both networks demonstrated excellent stability analysis results. In the scale-level network, the correlation stability coefficients (CS-coefficient) for all indices reached the maximum threshold: strength (0.75), expected influence (0.75), betweenness (0.75), and closeness (0.75). In the item-level network, coefficients were as follows: strength (0.750), expected influence (0.750), betweenness (0.516), and closeness (0.595).All centrality indices’ CS coefficients reached or exceeded the good standard of 0.5, indicating that the network’s main centrality indices and edge weight estimates possess strong stability, supporting the reliability of network structure interpretation based on these indices.

## 4 Discussion

This study systematically analyzed psychological health symptom association patterns among British university students through multilayer network analysis, constructing a comprehensive eight-dimensional symptom network including depression, anxiety, mania, sleep quality, stress, suicidal ideation, psychotic experiences, and loneliness. Results showed that at the scale level, depressive symptoms demonstrated prominence across all centrality indicators, establishing their core network position. In item-level analysis, death thoughts in depressive symptoms (PHQ_9), lack of companionship in loneliness symptoms (UCLA3_4), and frequency of suicidal thoughts (SBQ2) exhibited strongest bridge centrality, becoming key nodes connecting different symptom domains.

### 4.1 Network core position of depressive symptoms and its clinical significance

Depressive symptoms occupy an absolute core position in the psychological health network, consistent with recent network analysis research. A large-scale longitudinal study found that depressive symptoms showed highest strength centrality and expected influence in adolescent psychological health networks, becoming key nodes for activating other symptoms (25). A study of Dutch university students confirmed the network core role of depressive symptoms, particularly their importance in connecting anxiety symptoms (26). This study reveals refined connection patterns within depressive symptoms. The strong connection between PHQ_1 (lack of interest) and PHQ_2 (feeling depressed) (edge weight = 0.327) reflects the core role of negative cognition in cognitive triad theory (7). This finding echoes recent research (27) which found through large-scale network analysis that cognition-related depressive symptoms tend to have higher activation thresholds and easily form persistent symptom clusters once activated. PHQ_9 (death thoughts) ranked highest in bridge centrality analysis, having important clinical warning significance. Death thoughts, as a key bridge between depressive symptoms and suicidal ideation, provide important network indicators for early suicide risk identification. An ecological momentary assessment study found that within-day fluctuations in death thoughts could significantly predict subsequent changes in suicidal ideation, supporting dynamic mutual activation between symptoms in network theory (28).

### 4.2 Unique bridging role of loneliness

Loneliness functions as a bridge linking psychotic-like experiences, perceived stress, and suicidal ideation, providing empirical evidence for loneliness’s mechanistic role in psychological distress development. At symptom level, loneliness exhibits significant associations with psychotic-like experiences and perceived stress, while maintaining direct connection to suicidal ideation. Although this direct edge is weaker than the link between depressive symptoms and suicidal ideation, loneliness’s contribution to suicidal ideation is not fully mediated by depression. Instead, loneliness exerts a non-redundant, depression-independent effect on suicidal ideation, underscoring its supplementary significance in comprehensive suicide risk assessment. This finding is consistent with social pain theory (10), which emphasizes that loneliness triggers and maintains various mental health problems. An adolescent psychological health symptom network study confirmed loneliness’s significant bridge centrality, particularly in connecting internalizing and externalizing symptoms (18).

In item-level analysis, UCLA3_4 (lack of companionship) showed prominence across multiple bridge centrality indicators. This finding is consistent with a longitudinal study results that found that “lack of companionship” has unique roles in predicting subsequent development of depressive and anxiety symptoms (29). The two reverse-scored items UCLA3_19 and UCLA3_20 showed strongest internal connection, reflecting social support perception consistency, aligning with social support buffering model theoretical foundations (12).

### 4.3 Network positioning of suicidal ideation and risk assessment significance

This study provided important findings regarding suicidal ideation network positioning. SBQ2 (frequency of suicidal thoughts) and SBQ4 (future suicide possibility) showed significant performance in bridge strength and bridge expected influence indicators, demonstrating closest bridging relationships with depressive and loneliness symptoms. This finding provides network-level empirical support for interpersonal-psychological theory of suicide (19), which emphasizes thwarted belongingness, perceived burdensomeness, and acquired capability roles in suicide risk. The strong connection between SBQ1 (ever considered suicide) and PHQ_9 (thoughts of death) reflects conceptual overlap between suicidal ideation and death-related cognition in depressive symptoms. This finding is consistent with research results (20) which found that hopelessness and worthlessness in depressive symptoms typically show strong network connections with suicidal ideation. This study found direct network connections between suicidal ideation and loneliness, providing support which argued that social isolation may serve as an important bridge connecting other symptoms with suicidal ideation (30). A cognitive model of suicidal ideation emphasized social connectedness’s protective role, considering lack of belongingness as an important suicide risk predictor (19).

### 4.4 Network mechanisms of psychological health symptom comorbidity

This study revealed complex mechanisms of psychological health symptom comorbidity through dual-layer network analysis. At scale level, the strongest positive connection between anxiety and depression and the strong association between anxiety and stress reflect high comorbidity of internalizing disorder symptoms. This finding is consistent with tripartite model, which emphasizes negative affect’s role as common foundation for anxiety and depression (31). The significant negative association between sleep quality and depressive symptoms provides network evidence for bidirectional sleep-emotion model (32). This model suggests sleep problems are both consequences and maintaining factors of depressive symptoms. Recent longitudinal network studies (33) confirmed sleep quality’s important regulatory role in psychological health networks, finding that improving sleep quality can significantly reduce activation levels across entire symptom networks.

### 4.5 Application value of network analysis in precision intervention

Network analysis results provide scientific basis for precision intervention in psychological health. Based on network theory, interventions targeting high-centrality symptoms may produce cascade effects, influencing entire symptom network stability (34). Key symptom nodes identified--death thoughts in depressive symptoms, lack of companionship in loneliness, and frequency of thoughts in suicidal ideation--can serve as priority intervention targets. Network-based intervention strategies in randomized controlled trials found that cognitive behavioral therapy targeting high-centrality symptoms could produce better overall therapeutic effects than traditional treatments (35). Research (34) showed that network analysis can help identify individualized symptom patterns, providing guidance for developing personalized treatment plans.

It is critical to note that high centrality indices (such as strength and expected influence) identify nodes that are statistically prominent within the current network architecture. However, because this analysis is based on cross-sectional data, these results should be interpreted as identifying *potentially* relevant targets for intervention. Future longitudinal or experimental studies are essential to confirm the causal role of these symptoms in the activation and maintenance of the mental health network.

### 4.6 Limitations

This study has important limitations. First, cross-sectional design limits causal relationship inference. While network analysis can identify conditional independence relationships between symptoms, it cannot determine temporal sequence and causal direction of symptom activation. Second, unbalanced gender distribution (83% female) may affect result generalizability. Existing research shows that network structures of psychological health symptoms may have gender differences (36), so this study’s result applicability in male populations needs further verification. Finally, self-report measurements may involve common method bias, particularly when assessing subjective experiences such as loneliness and depressive symptoms.

## 5 Conclusion

In conclusion, this study delineates the core architecture of the mental health network in university students. Depressive symptoms occupy an unequivocally central position, while loneliness serves as a critical bridge to suicidal ideation. These findings suggest that while early identification of depressive symptoms may improve global functioning, addressing the ‘lack of companionship’ facet of loneliness may be pivotal in stemming suicide risk.

## Data Availability

The original contributions presented in the study are included in the article/Supplementary Material. Further inquiries can be directed to the corresponding author.
