# Supplementary material for: Multilayer network analysis of mental health symptoms in UK University students: association patterns of depression, loneliness, and suicidal ideation
Source: Front Psychiatry. 2026 Feb 27;17:1682965. doi: 10.3389/fpsyt.2026.1682965 (PMC12982387; doi:10.3389/fpsyt.2026.1682965)
Supplement: Supplementary file 1 [file SupplementaryFile1.pdf]

Supplementary Material

1 Supplementary Figures and Tables

1.1 Supplementary Figures

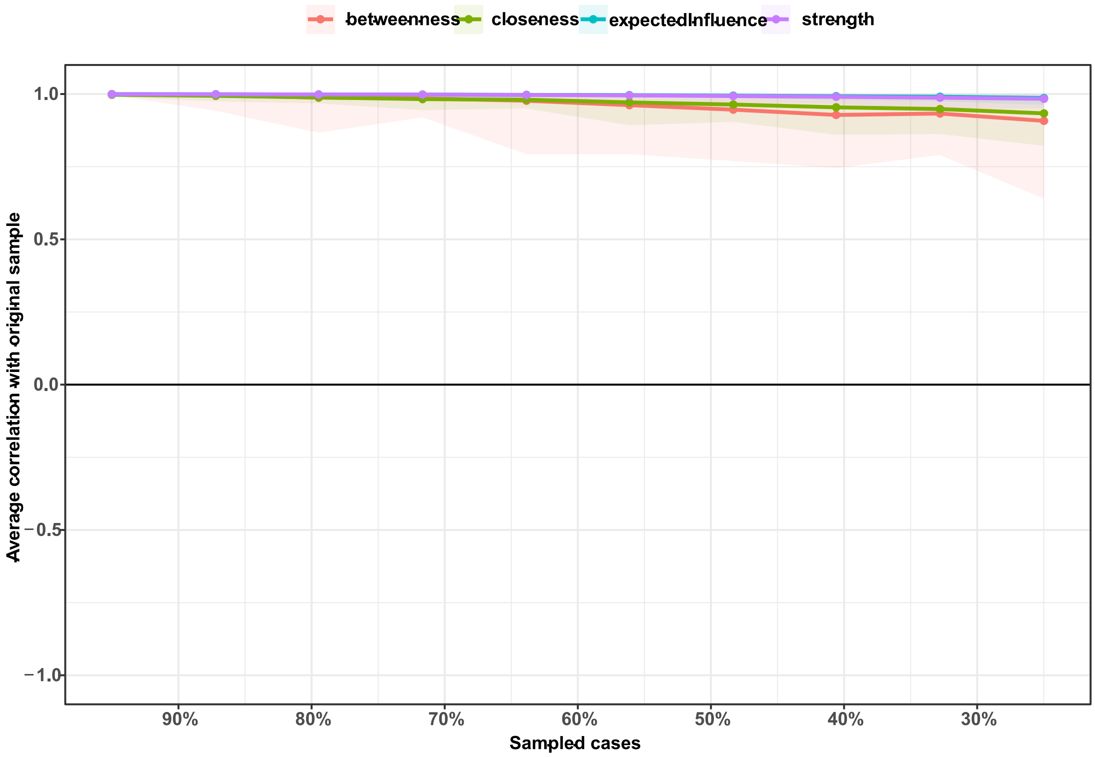

Supplementary Figure 1. The average correlation coefficients between selected cases and the full sample for the centrality indices of networks. Lines indicate the means and areas ranging from the 2.5th quantile to the 97.5th quantile

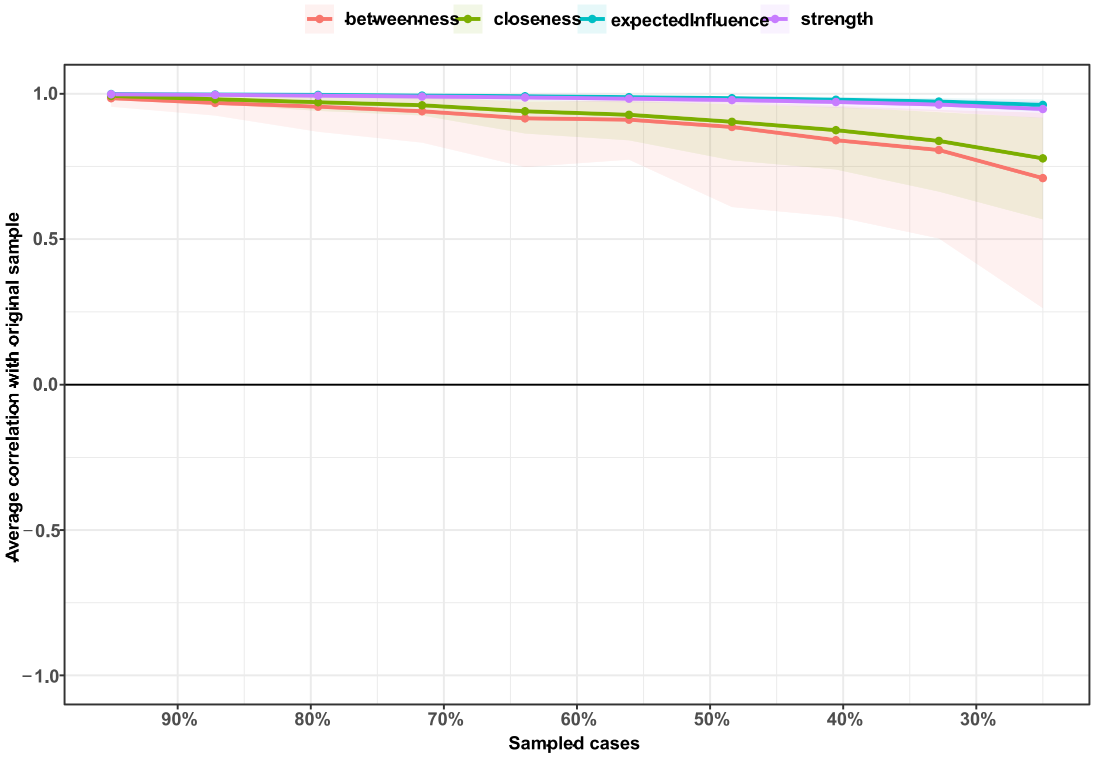

Supplementary Figure 2. The average correlation coefficients between selected cases and the full sample for the centrality indices of networks. Lines indicate the means and areas ranging from the 2.5th quantile to the 97.5th quantile
